# Embedded Computational Heart Model for External Ventricular Assist Device Investigations

**DOI:** 10.1007/s13239-022-00610-w

**Published:** 2022-03-15

**Authors:** Thomas Kummer, Simone Rossi, Stijn Vandenberghe, Stefanos Demertzis, Patrick Jenny

**Affiliations:** 1 Department of Mechanical and Process Engineering, ETH Zurich, Zurich, Switzerland; 2 Mathematics Department, University of North Carolina, Chapel Hill, NC USA; 3 Cardiovascular Engineering, Cardiocentro Ticino, Lugano, Switzerland; 4 Faculty of Biomedical Sciences, Università della Svizzera italiana, Lugano, Switzerland; 5 Cardiac Surgery & Cardiovascular Engineering, Cardiocentro Ticino, Lugano, Switzerland

**Keywords:** External ventricular assist device, Direct cardiac contractor, Medical device development, Three dimensional heart modeling, Active strain actuation, Lumped parameter circulation, Hydraulic network model

## Abstract

**Purpose:**

External cardiac assist devices are based on a promising and simple concept for treating heart failure, but they are surprisingly difficult to design. Thus, a structured approach combining experiments with computer-based optimization is essential. The latter provides the motivation for the work presented in this paper.

**Methods:**

We present a computational modeling framework for realistic representation of the heart’s tissue structure, electrophysiology and actuation. The passive heart tissue is described by a nonlinear anisotropic material law, considering fiber and sheetlet directions. For muscle contraction, an orthotropic active-strain model is employed, initiated by a periodically propagating electrical potential. The model allows for boundary conditions at the epicardium accounting for external assist devices, and it is coupled to a circulation network providing appropriate pressure boundary conditions inside the ventricles.

**Results:**

Simulated results from an unsupported healthy and a pathological heart model are presented and reproduce accurate deformations compared to phenomenological measurements. Moreover, cardiac output and ventricular pressure signals are in good agreement too. By investigating the impact of applying an exemplary external actuation to the pathological heart model, it shows that cardiac patches can restore a healthy blood flow.

**Conclusion:**

We demonstrate that the devised computational modeling framework is capable of predicting characteristic trends (e.g. apex shortening, wall thickening and apex twisting) of a healthy heart, and that it can be used to study pathological hearts and external activation thereof.

## Introduction

Advanced heart failure is currently treated most successfully by heart transplantation. However, donor organ availability is very limited. Thus, over the past decades, ventricular assist devices (VADs) have become the standard alternative medical therapy.^25^ Usually, turbodynamic flow pumps support or replace left ventricular functions; and in severe cases, both heart chambers. Some patients receive a VAD only as a bridge to heart transplant, whereas for some patients it is their destination therapy. Over the years, many products have been invented; some work in a pulsatile manner, but most of them provide a more or less continuous flow. What all of the nowadays implanted devices have in common is that they remove blood from a heart chamber and deliver it into the ascending great vessels. Downsides of existing VADs are the surgical opening of anatomical structures of a closed system, direct contact with the blood stream and unnatural temporal actuation. To avoid these shortcomings, other concepts have been suggested, e.g. to use an inflating balloon in the right ventricle^15^ or the intrapericardial cavity,^11,17,21^ two contracting rings around the heart,^33^ inflatable diaphragms on the heart’s surface,^20,31,48^ curvature inverting devices,^27,28,30^ a twisting system^7^ and the criscone device.^28^

**Figure 1:**
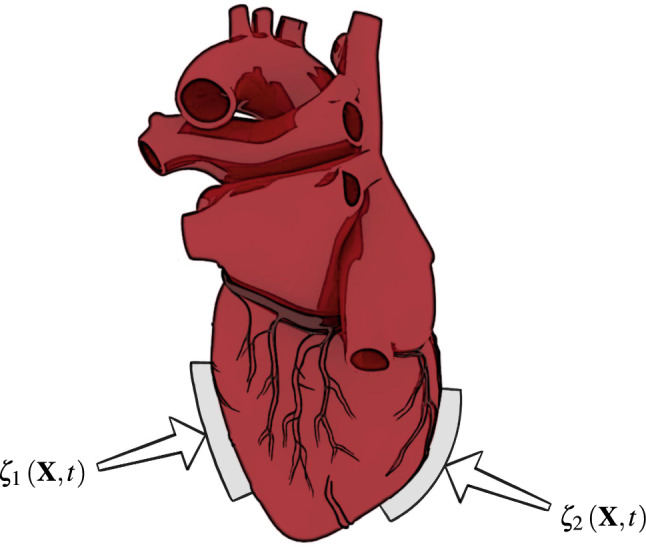
Force patches ${\varvec{\zeta }}_1\left( {\mathbf{X}},t\right)$ and ${\varvec{\zeta }}_2\left( {\mathbf{X}},t\right)$ attached to the outer heart surface for actuation. The three-dimensional human heart model is from Ref. [1].

Here, an external mechanical actuation concept, inspired by open cardiac massage and previous work,^16,41^ is considered. Similar as the surgeon’s palms, two opposed force patches periodically compress the heart and thus enhance the circulation. Before such devices can be used even for short term applications, such as during heart surgeries, numerous challenges need to be addressed. In addition to the difficulties in achieving desired flow rates and pressure differences, the consequences of attaching force patches directly on the epicardium are unclear. As illustrated in Fig. 1, epicardial force patches may lead to undesired deformations of the papillary muscles and heart valves, tissue fatigue, squeezing of the coronary arteries underneath the patches, and critical stress peaks. Understanding how these factors influence cardiac function are important to assess the efficacy of the device.

The main focus of this paper lies on the development of a computational framework suited to investigate external application of force patches while gaining a better understanding of cardiac mechanics. Investigating the impact of VADs on the heart function using experiments can be difficult, if not impossible, and expensive. On the other hand, computer simulations of the heart allow to study a variety of pathologies more easily. For example, one can use computational heart models reflecting coronary artery diseases, electrophysiological abnormalities or pressure-volume anomalies. Moreover, a computational model allows for optimization studies; objectives are e.g. cardiac output, overall contraction pattern, stress peaks or the right ventricle’s vulnerability. By adjusting force patches as functions of space and time, the desired flow rates and pressures can be imposed. The center piece of the computational framework presented in this paper is based on the finite element library LifeV, which implements a detailed model of the heart structure accounting for the highly anisotropic muscle, mechanical and electrical behavior. A Holzapfel–Ogden model^8,14^ is employed, and required fiber- and sheetlet directions are generated according to Refs. 43, 52. Rigid external force patches are modeled using time-dependent non-homogeneous Dirichlet boundary conditions. Muscle activation, which is modeled by an orthotropic active strain formulation^2,29,38,43,44^ and causes the cardiomyocytes to contract, is triggered by an electrical wave.^3^ Although similar frameworks have been published,^9,12,13,34,37,47,50^ activation has not been described by active strain nor has the effect of force patches been studied. Finally, in order to calculate blood pressure in the two ventricles, which is required for properly predicting deformations, the finite element model is coupled to a simplified hydraulic network accounting for pulmonary and systemic circulations.^6,46^ For the heart valves a novel pressure gradient dependent model is proposed.

The paper is organized as follows. In Embedded Heart Model and Governing Equations section we describe the governing equations of the individual sub-models for electrophysiology, activation, structure mechanics and the lumped parameter circulation. The computational framework, that is, the numerical solution algorithms and coupling thereof, is presented in Methods: Computational Framework section. Numerical results are shown in Validation and Results section; first of a healthy heart and then of one suffering from coronary heart disease. Moreover, simulation results with the pathological heart model assisted by two force patches and a parameter sensitivity study are presented. The paper closes with a discussion and conclusions in Discussion and Conclusion section.

## Embedded Heart Model and Governing Equations

### Mechanical Response of the Myocardium

Let $\varOmega _0 \subset {\mathbb {R}}^3$ be the undeformed structure configuration of the heart with coordinates denoted by ${\mathbf{X}}$, and ${\mathbf{x}}$ being the current position of the deformed body $\varOmega \subset {\mathbb {R}}^3$. The undeformed boundary $\partial \varOmega _0$ is divided into $\varGamma _{\text{D}}$ with Dirichlet boundary conditions, $\varGamma _{\text{N}}$ with Neumann boundary conditions and $\varGamma _{\text{R}}$ with Robin boundary conditions. Inertial and body forces (due to gravity) are negligible compared to other forces^51^ and thus are ignored here. The resulting quasi-static boundary value problem reads

2.1 $$\begin{array}{ll} {\nabla }\cdot {{\mathbf{P}}}({\mathbf{u}}) = {\mathbf{0}}, &\text {in } \varOmega _0 \text { with} \\{\mathbf{u}} = {\mathbf{u}}_{\text{D}},&\text {on } \varGamma _{\text{D}}, \\{\mathbf{P}} \cdot {\varvec{\nu }} = {\mathbf{p}}_{\text{N}},&\text {on } \varGamma _{\text{N}} \text { and} \\{\mathbf{P}} \cdot {\varvec{\nu }} + \alpha \, {\mathbf{u}} = {\mathbf{0}},&\text {on } \varGamma _{\text{R}}, \end{array}$$

where ${\mathbf{u}}={\mathbf{x}}-{\mathbf{X}}$ is the displacement, ${\mathbf{P}}$ the first Piola-Kirchhoff stress tensor, ${\varvec{\nu }}$ the outward surface normal in the reference configuration and ${\mathbf{u}}_{\text{D}}$ and ${\mathbf{p}}_{\text{N}} = p \, {\varvec{\nu }}$ Dirichlet and Neumann boundary conditions, respectively. Note that $\nabla$ is operated with respect to ${\mathbf{X}}$ in the reference configuration.

The myocardium is considered hyperelastic, and therefore its mechanical response can be fully characterized by a scalar-valued strain-energy function $\varPsi$. We consider an orthotropic description of the myocardium given by the Holzapfel-Ogden constitutive law,^14^ which leads to

2.2 $$\begin{aligned} \varPsi = & {} \frac{a}{2b} e^{b({I}_1 - 3)} + \sum _{i \in \{f,s\}} \frac{a_i}{2b_i} \left( e^{b_i(I_{4i} - 1)^2} -1 \right) \nonumber \\ & \quad + {} \frac{a_{fs}}{2b_{fs}} \left( e^{b_{fs}(I_{8fs})^2} - 1 \right) \end{aligned}$$

for the passive strain-energy function with the four invariants $I_1 = {\text{tr}} \left( {\mathbf{C}}\right)$, $I_{4{{f}}} = {\mathbf{f}}\cdot {\mathbf{f}}$, $I_{4s} = {\mathbf{s}}\cdot {\mathbf{s}}$ and $I_{8fs} = {\mathbf{f}}\cdot {\mathbf{s}}$, which are functions of the right Cauchy-Green tensor ${\mathbf{C}} = {\mathbf{F}}^T {\mathbf{F}}$ and the fiber and sheetlet directions ${\mathbf{f}}$ and ${\mathbf{s}}$, respectively. The constants *a*, *b*, $a_i$ and $b_i$ $\left( i \in \{f,s,fs\}\right)$ were found by fitting experimental data.^5^ The stress tensor is defined by the relation ${\mathbf{P}} = \frac{\partial \varPsi }{\partial {\mathbf{F}}}$, in which ${\mathbf{F}} = {\nabla }\,{\mathbf{x}} = {\mathbf{I}} + {\nabla }\,{\mathbf{u}}$ is the deformation gradient tensor.

Because numerical experiments have shown that the active-strain formulation is capable of reproducing appropriate contraction patterns, we follow such an approach.^29,43^ Assuming the deformation gradient admits a multiplicative decomposition, ${\mathbf{F}} = {\mathbf{F}}_E {\mathbf{F}}_A$, in which ${\mathbf{F}}_E$ is the passive, elastic deformation and ${\mathbf{F}}_A$ is an active deformation described by the expression

**Figure 2:**
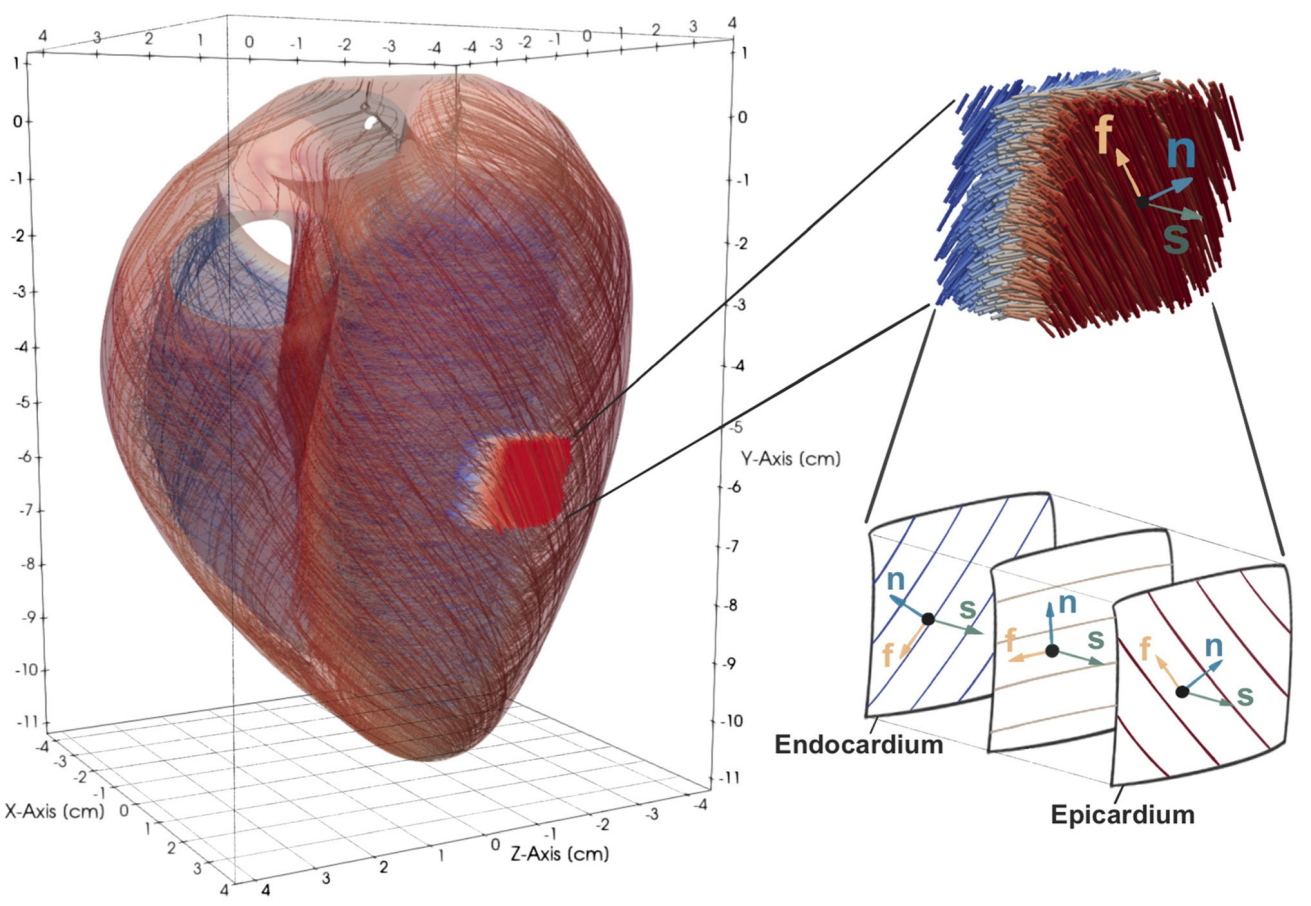
Enlarged section of the heart tissue with fiber- and sheeltlet vectors ${\mathbf{f}}$ and ${\mathbf{s}}$, respectively; ${\mathbf{n}}={\mathbf{f}}\times {\mathbf{s}}$. Note that the direction of the fibers depends on the layer.

2.3 $$\begin{aligned} {\mathbf{F}}_A = {\mathbf{I}} + \gamma _{f} {\mathbf{f}}_0 \otimes {\mathbf{f}}_0 + \gamma _{s} {\mathbf{s}}_0 \otimes {\mathbf{s}}_0 + \gamma _{n} {\mathbf{n}}_0 \otimes {\mathbf{n}}_0, \end{aligned}$$

in which ${\mathbf{I}}$ is the identity tensor. The active-strain functions $\gamma _f$, $\gamma _s$ and $\gamma _n$ provide information on the contractile strains. Notice that ${\mathbf{f}}_0$, ${\mathbf{s}}_0$ and ${\mathbf{n}}_0$ are orthogonal directions in the reference configuration; see Fig. 2. Given the decomposition of the deformation gradient tensor into an active and elastic component, the active strain formulation assumes that the strain energy can be expressed by Eq. (2.2), where the invariants $I_{4{{f}}}$, $I_{4s}$, $I_{8fs}$ and ${I}_1$ have to be replaced by

2.4 $$\begin{aligned} I_{4{{f}}}^E= & {} I_{4{{f}}}\left( \gamma _f+1\right) ^{-2}, \end{aligned}$$

2.5 $$\begin{aligned} I_{4s}^E= & {} I_{4s}\left( \gamma _s+1\right) ^{-2},\end{aligned}$$

2.6 $$\begin{aligned} I_{8fs}^E= & {} I_{8fs}\left( \gamma _f+1\right) ^{-1}\left( \gamma _s+1\right) ^{-1}\ \ \ \ \text {and}\end{aligned}$$

2.7 $$\begin{aligned} {I}^E_1= & {} \Bigg [ 1 - \frac{\gamma _n\left( \gamma _n+2\right) }{\left( \gamma _n+1\right) ^2} \Bigg ] {I}_1 + \Bigg [ \frac{\gamma _n\left( \gamma _n+2\right) }{\left( \gamma _n+1\right) ^2} - \frac{\gamma _f\left( \gamma _f+2\right) }{\left( \gamma _f+1\right) ^2} \Bigg ] I_{4{{f}}} \nonumber \\ & \quad + {} \Bigg [\frac{\gamma _n\left( \gamma _n+2\right) }{\left( \gamma _n+1\right) ^2} - \frac{\gamma _s\left( \gamma _s+2\right) }{\left( \gamma _s+1\right) ^2} \Bigg ] I_{4s}, \end{aligned}$$

respectively.

### Active Strain Dynamics

In Eq. (2.3), the active part of the deformation gradient ${\mathbf{F}}_A$ is determined by the three active-strain functions $\gamma _f$, $\gamma _s$ and $\gamma _n$. Appropriate definitions for these have been derived in Ref. [43]. Thus, we assume the active strain in fiber direction $\gamma _f$ satisfies

2.8 $$\begin{aligned} \mu _A c^2 \; \frac{{\text{d}}\gamma _f}{{\text{d}}t} = \, \alpha _A (c-c_0)^2R_{{FL}}(I_{4{{f}}}) + \frac{2 I_{4{{f}}}}{(1+\gamma _f)^3} - 2 I_{4{{f}}}|_{c=c_0}, \end{aligned}$$

where *c* is the calcium concentration coming from electrophysiology, $I_{4{{f}}}$ is the fiber elongation from mechanics, $\mu _A$ and $\alpha _A$ are constants and $R_{{FL}}$ is the sarcomere force–length relationship, which can be found in Ref. [43]. The two other active strain functions $\gamma _s$ and $\gamma _n$ are determined by imposing volume conservation, i.e., $\det ({\mathbf{F}}_A) = 1$, and are functions of $\gamma _f$ and an orthotropic parameter $\kappa$, i.e.,

2.9 $$\begin{aligned} \gamma _n = \kappa \gamma _f \quad \text {and} \quad \gamma _s = \frac{1}{(1+\gamma _f)(1+\gamma _n)} - 1. \end{aligned}$$

### Electrophysiology

Before any contraction of the heart muscle starts, the sinoatrial node (SA-node) initiates a wave of electrical depolarization propagating from the right atrium to the left atrium and to the atrioventricular node (AV-node). The AV-node delays the signal by approximately 0.12 s, before it propagates through the ventricular muscles. From the AV node, the electric signal travels through the fast cardiac conduction system to quickly activate the endorcardial surface in multiple locations and ensure a uniform ventricular contraction. To reproduce the signal after the AV-node, we use the monodomain equations combined with the minimal model for human myocardial action potential.^3^ In the reference domain $\varOmega _0$ the monodomain equations read

2.10 $$\begin{aligned}&J\,\chi \Big ( C_{{m}} \frac{{\text{d}}V}{{\text{d}}t} + I_{\text{ion}}(V,{\mathbf{w}},{\mathbf{c}},{\mathbf{F}}) \Big ) \\&\quad - {\nabla }\cdot \Big ( J\,{\mathbf{F}}^{-1}{\mathbf{G}} {\mathbf{F}}^{-T} \, {\nabla }\,V \Big ) = I_{\text{app}} \quad \text {in } \varOmega _0 \text { with} \\&\Big ( {\mathbf{F}}^{-1}{\mathbf{G}} {\mathbf{F}}^{-T} \, {\nabla }\,V \Big ) \cdot {\varvec{\nu }}= 0 \quad \text {on } \partial \varOmega _0 \text { and} \\&\frac{{\text{d}}{\mathbf{w}}}{{\text{d}}t} - {\mathbf{r}} (V, {\mathbf{w}}) = 0 \quad \text {in } \varOmega _0, \end{aligned}$$

in which *V* is the transmembrane potential, the vector ${\mathbf{w}}$ represents the gating variables, ${\mathbf{c}}$ is the vector of ionic concentrations, $I_{\text{app}}$ is an externally applied density current, $I_{\text{ion}}$ and ${\mathbf{r}}$ link the two equations, ${\mathbf{G}}$ is the spatial (in the deformed configuration) anisotropic conductivity tensor, depending on the fiber and sheetlet directions ${\mathbf{f}}_0$ and ${\mathbf{s}}_0$, $\chi$ and $C_m$ are constants, ${\mathbf{F}}$ is the deformation gradient and $J=\det ({\mathbf{F}})$ is its determinant.

### Fiber and Sheetlet Directions

While DT-MRI data for reconstructing fiber fields are available, information on the collagen sheets is usually unavailable. Here, both fields are reconstructed using a rule-based approach.^8,43,52^ Assuming the sheetlet field ${\mathbf{s}}_0$ to be divergence- and curl-free, ${\mathbf{s}}_0$ can be derived from a scalar potential $\phi$ as ${\mathbf{s}}_0 = {\nabla }\,\phi$. Taking the divergence of the equation, a Laplace-equation for the potential $\phi$ is obtained, that is,

2.11 $$\begin{aligned} \nabla ^2\,\phi = 0 \qquad \text {in} \ \varOmega _0 \end{aligned}$$

with Dirichlet boundary conditions $\phi = g \ \text {on} \ \varGamma _{\text{D}}$ and Neumann boundary conditions $\frac{\partial \phi }{\partial {\varvec{\nu }}} = h \ \text {on} \ \varGamma _{\text{N}}$. Having solved Eq. (2.11) inside the undeformed myocardium for $\phi$, the sheetlet vector is ${\mathbf{s}}_0 = {{\nabla }\,{\phi }}/{||{\nabla }\,{\phi }||}$ and points from the endocardium toward the epicardium. Next, consider the left ventricular centerline ${\mathbf{k}}$, pointing from the apex to the base. Its projection on the plane orthogonal to ${\mathbf{s}}_0$ is ${\mathbf{k}}_p = {\mathbf{k}} - \left( {\mathbf{k}} \cdot {\mathbf{s}}_0 \right) {\mathbf{s}}_0$. Figure 2 shows an enlarged section of the heart tissue with fiber- and sheeltlet vectors ${\mathbf{f}}$ and ${\mathbf{s}}$, respectively; ${\mathbf{n}}={\mathbf{f}}\times {\mathbf{s}}$. Note that the direction of the fibers depend on the layer. A preliminary, horizontal fiber field is orthogonal to both ${\mathbf{s}}_0$ as well as ${\mathbf{k}}_p$ and reads $\tilde{{\mathbf{f}}}_0 = {\mathbf{s}}_0 \times {\mathbf{k}}_p / ||{\mathbf{k}}_p||$. Ultimately, the actual fiber field is ${\mathbf{f}}_0 = {\mathbf{R}}_{{\mathbf{s}}_0} \left( \theta \right) \tilde{{\mathbf{f}}}_0$, where ${\mathbf{R}}_{{\mathbf{s}}_0}$ is a rotation matrix dependent on the fiber angle $\theta$. More details about building ${\mathbf{R}}_{{\mathbf{s}}_0}$ can be found in Ref. [43], where it also is shown that computationally obtained fiber fields are in good agreement with dissections.^45^

### Circulation

**Figure 3:**
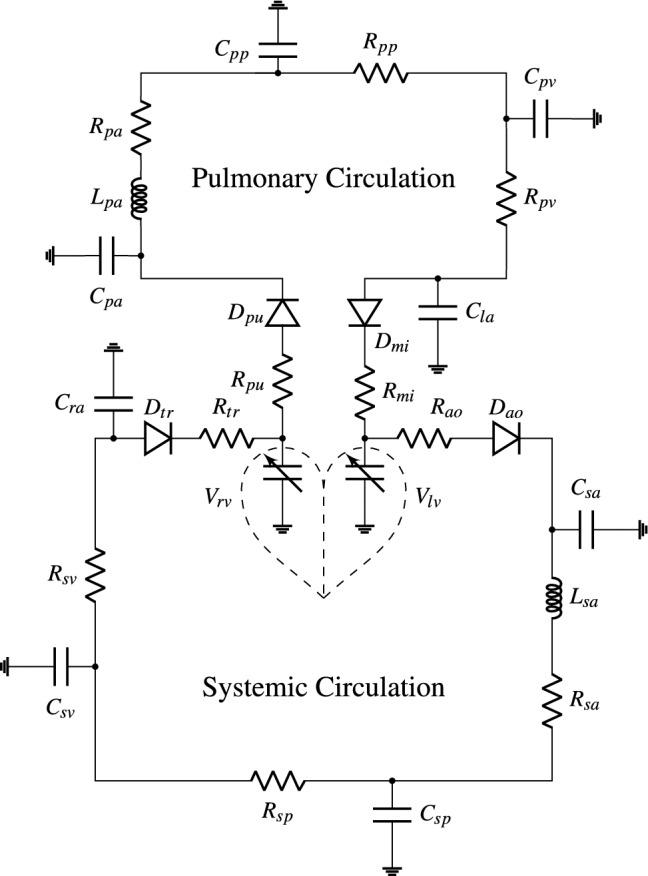
Lumped parameter model of the blood flow in the human body. The pulmonary circulation, where blood is oxygenated, is drawn at the top. At the bottom, the systemic circulation is shown, which delivers the oxygen to the body’s tissue. This model is an adaption from Ref. [6, 19]. The meaning of the symbols is shown in Table 1.

The body circulation is represented by the circuit depicted in Fig. 3, which is adapted from Ref. [6, 19, 46]. The driving force of the circulation is exerted by both the right and left heart chambers. This force is a boundary condition in Eq. (2.1) and can be divided into pressure, viscous and inertial forces. Because forces due to inertia and wall shear stress inside the heart are much smaller than the pressure force,^36^ we neglect their contribution. Moreover, because in both ventricles the dynamic blood pressure is much smaller than the static pressure variations during a heart beat, we assumed a spatially constant pressure field.^9,10,24^

**Table 1:** Meaning of subscripts of compliances *C* [mL mmHg^−1^], resistances *R* [mmHg s mL^−1^], inertances *L* [mmHg mL s^−2^] and diodes *D* [−] in the body circulation found in Fig. 3. The variable compliances account for changes of left and right ventricular volumes $V_{\text{lv}}$ and $V_{\text{rv}}$, respectively.

Subscript	Meaning
sa	Systemic arteries
sv	Systemic veins
sp	Systemic peripheries
pa	Pulmonary arteries
pv	Pulmonary veins
pp	Pulmonary peripheries
ao	Aortic valve
mi	Mitral valve
pu	Pulmonary valve
tr	Tricuspid valve
ra	Right atrium
la	Left atrium

Ventricles are drawn as variable capacitors and stand for the three-dimensional finite element model of the heart. Both the pulmonary and systemic circulations include three resistances, three capacitances and an inertance. Each of the heart valves is modeled by a resistance and a diode, and both atria are modeled as a capacitance. The dependent variables are pressure values *p* at the nodes, from which the flow rates can be calculated. Lumped parameter models as used here obey Kirchhoff’s circuit laws and are by construction conservative. Thus, the sum of all flow rates $q_{i-j}$ from node *i* to its connected nodes *j* is zero, i.e.,

2.12 $$\begin{aligned} \sum _j q_{i-j} = 0. \end{aligned}$$

The resistance *R* between two nodes *i* and *j* is the ratio of pressure difference $p_i - p_j$ and flow rate $q_{i-j}$, which results in

2.13 $$\begin{aligned} q_{i - j} = \frac{p_i - p_j}{R}. \end{aligned}$$

Inertia in both aorta and pulmonary artery is modeled by the inertences $L_{\text{sa}}$ and $L_{\text{pa}}$, respectively, i.e., the corresponding pressure difference is a function of temporal flow rate change according to $p_i - p_j=L\ dq_{i - j}/dt$, which leads to

2.14 $$\begin{aligned} \frac{{\text{d}}q_{i - j}}{{\text{d}}t} = \frac{p_i - p_j}{L}. \end{aligned}$$

Blood vessel elasticity is modeled by compliances *C* between a certain node *i* and the ground, from which one obtains

2.15 $$\begin{aligned} q_{i-\infty } = C \, \frac{{\text{d}}p_i}{{\text{d}}t}. \end{aligned}$$

To account for the heart valves, a new diode model is proposed here. In an ideal setting, diodes are open for a positive pressure gradient and closed otherwise. However, heart valves are not ideal diodes and therefore it is suggested here to model them as

2.16 $$\begin{aligned} q_{i-j} = D\ \frac{p_i - p_j}{R}, \end{aligned}$$

that is, as a compartment consisting of a resistance *R* and a diode $D = D \left( p_i, p_j, t \right) \in \left[ 0,1\right]$. In other words, a valve is described as a temporally varying resistance, that is, if it is open, then $D=1$, while $D=0$ if it is closed. The diode dynamics is defined by

2.17 $$\begin{aligned} D = {\left\{ \begin{array}{ll} D_u, &{}\hbox { if}\ D_u < D_m, \\ D_l, &{}\hbox { if}\ D_l > D_m, \\ D_m, &{}\text {otherwise}, \end{array}\right. } \end{aligned}$$

and the three simple intermediate differential equations

2.18 $$\begin{aligned} \frac{{\text{d}}^2 D_m}{{\text{d}}t^2}&= \lambda \frac{p_i - p_j}{|p_i - p_j|}, \end{aligned}$$

2.19 $$\begin{aligned} \frac{{\text{d}}D_u}{{\text{d}}t}&= \gamma _u (1 - D_u) \quad \text {and} \end{aligned}$$

2.20 $$\begin{aligned} \frac{{\text{d}}D_l}{{\text{d}}t}&= -\gamma _l D_l \end{aligned}$$

with the constants $\lambda \, [s^2]$, $\gamma _u \, [s]$ and $\gamma _l \, [s]$, which are used to adjust the time required to open and close a valve. The state of a valve is determined by Eq. (2.18), except if *D* is in the vicinity of 0 (closed) or 1 (open). Then the respective Eqs. (2.18) and (2.19) determine how the valve state changes. The purpose of this simple valve model is adjustability of the time it takes to open or close as well as to ensure time continuous signals. Note, however, that the focus of this study is not on valve models.

The resistance of the outflow valves additionally depends on the ventricular pressure values.^6^ For example, the resistance of the aortic valve is $R_{\text{ao}} = k_{\text{lv}} \, p_{\text{lv}}$, where $k_{\text{lv}}$ is a constant parameter. Moreover, when the heart valves open, ventricular volume decreases and thus the contraction is no more considered isometric. An instantaneous ventricular pressure $p_{\text{lv,inst}}$ is obtained as the difference between the isometric pressure $p_{\text{lv}}$ and viscous losses,^6^ namely, $p_{\text{lv,inst}} = p_{\text{lv}} - R_{\text{ao}} \, q_{\text{ao}}$. The same is done for the instantaneous pressure in the right ventricle.

The link between the circuit and the mechanics is established *via* pressure and volume of the two nodes representing the ventricles and will be explained in more detail later. Changes of left and right ventricular volumes are described as

2.21 $$\frac{{\text{d}}V_l}{{\text{d}}t} = q_{\text{mi}} - q_{\text{ao}} \quad {\text{and}}$$

2.22 $$\begin{aligned}&\frac{{\text{d}}V_r}{{\text{d}}t} = q_{\text{tr}} - q_{\text{pu}}, \end{aligned}$$

where $q_{mi}$, $q_{\text{ao}}$, $q_{\text{tr}}$ and $q_{\text{pu}}$ denote the flow rates through the four heart valves.

## Methods: Computational Framework

The overall framework has been implemented by using the parallel finite element library LifeV (http://www.lifev.org). Initial data and model parameters can be found in the Tables 3,  4 and 5.

### Finite Element Discretization for Electromechanics

Equation (2.1) is solved with a Galerkin finite element method. Tetrahedral elements with quadratic shape functions are employed for all electromechanical fields and a parallel version of the generalized minimal residual method (GMRES) is used to deal with the resulting system of linear equations. Solution of the non-linear problem is achieved with Newton’s method.

Incompressibility of the heart tissue is enforced by decomposing the strain energy function into an isochoric and a volumetric part, i.e.,

3.1 $$\begin{aligned} \varPsi = W_{A}\left( {\bar{I}}_1, I_{4,f}, I_{4,s}, I_{8,fs}\right) + W_{V}\left( I_3\right) , \end{aligned}$$

where the volumetric penalty function $W_{V}\left( I_3\right)$ is added to Eq. (2.2) and only depends on the invariant $I_3 = J^2 = (\det F)^2$. The function $W_{V} = \frac{K}{4}\left[ (J-1)^2 + ln^2J\right]$ with bulk modulus $K = 350 \; {\text{kPa}}$ is used in this work. For the term $W_{A}\left( {\bar{I}}_1, I_{4,f}, I_{4,s}, I_{8,fs}\right)$ in Eq. (3.1) to be isochoric, all four invariants in the argument list must be isochoric as well. However, significant changes only concern the first invariant $I_1$,^42^ which is replaced in Eq. (2.2) by ${\bar{I}}_1 = J^{-\frac{2}{3}} \, I_1$. Coefficients for the isochoric part can be found in Table 2.

**Table 2:** Top: material law parameters used in Eq. (2.2) taken from Ref. [14]. Bottom: initial electrophysiology data ($w_0$, $w_1$, $w_2$ and $w_3$) and monodomain model parameters.^43^

$a = 0.333 \; {\text{kPa}}$	$a_f = 18.535 \; {\text{kPa}}$
$a_s = 2.564 \; {\text{kPa}}$	$a_{fs} = 0.417 \; {\text{kPa}}$
$b = 9.242 \;$	$b_f = 15.972 \;$
$b_s = 10.446 \;$	$b_{fs} = 11.602 \;$
$w_0 = 0.0$	$w_1 = 0.986002$
$w_2 = 0.6818$	$w_3 = 0.02155$
$C_m = 1 \; {\mu\text{F}/\text{cm}^2}$	$\chi _m = 1400 \; \text{cm}^{-1}$
$\sigma _f = 10 \; {{\text{k}}\Omega ^{-1}{\text{cm}}^{-1}}$	$\sigma _s = 3 \; {{\text{k}}\Omega ^{-1}{\text{cm}}^{-1}}$

Active strain evolution in Eq. (2.8) is solved explicitly in time and its dependency on the prestretch is removed by a Taylor-expansion,^43^ i.e.,

3.2 $$\begin{aligned}&\mu _A c^2 \frac{{\text{d}}\gamma _f}{{\text{d}}t} = \alpha _A (c-c_0)^2 R_{{FL}}(I_{4{{f}}})\\&\quad + \sum _{j=1}^5 (-1)^j(j+1)(j+2)I_{4{{f}}}\gamma _f^j. \end{aligned}$$

Active strain measures $\gamma _f$, $\gamma _s$ and $\gamma _n$ are spatially discretized in the same manner as the structural mechanics; however, they are scalar fields. Time discretization of Eq. (3.2) is done by a forward Euler scheme. Active strain model coefficients are $\alpha _A = -6.25 \; {\mu {\text{M}}^{-2}}$, $\mu _A = 5000 \; {{\text{s}} \mu {\text{M}}^{-2}}$, $c_0 = 0.2155$ and $\kappa = 4$.

Equation (2.10) is solved by a semi-explicit method.^38^ Therefore, the gating variables ${\mathbf{w}}$ are updated explicitly by a forward Euler scheme first. The transmembrane potential *V* is determined in a second step by ionic current interpolation (ICI).^32^ Spatial discretization is the same as for activation and the mechanics. Ionic cell model (Minimal Model) parameters are found in Ref. [3] for the myocardium and monodomain model parameters as well as electrophysiology initial data in Table 2.

### Ordinary Differential Equations for Circulatory Model

The hydraulic circuit depicted in Fig. 3 is described by Eqs. (2.12)–(2.20). Note that the circuit model is not an integral part of the finite element library LifeV, i.e., it had to be implemented separately. Coupling with the heart model is described in “Coupling Mechanics and Circulation” section. By combining Eqs. (2.12)–(2.16), the system

3.3 $${\mathbf{M}}\frac{{\text{d}}{\varvec{\omega }}}{{\text{d}}t} + {\mathbf{A}} {\varvec{\omega }}= {\varvec{\varphi }}$$

of ordinary differential equations is obtained, where the vector of unknowns, ${\varvec{\omega }}^T = \left[ {\mathbf{b}}^T \quad {\mathbf{q}}^T\right]$, consists of a vector ${\mathbf{b}}$, which stores nodal pressure values, and of a vector ${\mathbf{q}}$ storing flow rates between neighboring nodes. Time discretization is done by a backward Euler scheme leading to

3.4 $$\begin{aligned} \left( \frac{{\mathbf{M}}}{\tau _c} + {\mathbf{A}} \right) {\varvec{\omega }}^{n+1} = {\varvec{\varphi }} + \frac{{\mathbf{M}}}{\tau _c} {\varvec{\omega }}^{n} \end{aligned}$$

with the unknown solution ${\varvec{\omega }}^{n+1}$ at the new time $t^{n+1}$, the previously computed solution ${\varvec{\omega }}^{n}$ at the old time $t^n$ and the time step size $\tau _c=t^{n+1}-t^n$. Considering Eqs. (2.17)–(2.20), which determine whether a heart valve is open or closed, it can be seen that the valve state *D* is a function of the pressure difference across the heart valve. This implies that matrices ${\mathbf{M}}$ and ${\mathbf{A}}$ as well as the vector ${\varvec{\varphi }}$ depend on ${\varvec{\omega }}$. In order to retain an implicit scheme, a fix-point iteration algorithm is employed, which reads

3.5 $$\begin{aligned} \left( \frac{{\mathbf{M}}^k}{\tau _c} + {\mathbf{A}}^k \right) {\varvec{\omega }}^{n+1,k+1} = {\varvec{\varphi }}^k + \frac{{\mathbf{M}}^k}{\tau _c} {\varvec{\omega }}^{n}, \end{aligned}$$

where *k* is the step number, ${\mathbf{M}}^k = {\mathbf{M}}\left( {\varvec{\omega }}^{n+1,k}\right)$, ${\mathbf{A}}^k = {\mathbf{A}}\left( {\varvec{\omega }}^{n+1,k}\right)$ and ${\varvec{\varphi }}^k = {\varvec{\varphi }}\left( {\varvec{\omega }}^{n+1,k}\right)$. At the initial step $k = 0$, ${\varvec{\omega }}^{n+1,0}$ is set to ${\varvec{\omega }}^{n}$. Fix-point iterations are carried out as long as

3.6 $$\begin{aligned} \frac{||{\varvec{\omega }}^{n+1,k+1} - {\varvec{\omega }}^{n+1,k}||_2}{||{\varvec{\omega }}^{n+1,k}||_2} > \varepsilon _c, \end{aligned}$$

where $\varepsilon _c = 10^{-6}$ is the desired relative change from one to the next iteration step. Parameters for various electric/hydraulic elements can be found in Table 3.

**Table 3:** Body circulation parameters for compliances, resistances and inertances found in Fig. 3. The values are required in Eqs. (2.13)–(2.15) and are adapted from Ref. [6, 46].

Compliance [mL mmHg^−1^]	Resistance [mmHg s mL^−1^]	Inertance [mmHg mL s^−2^]
${C_{\text{sa}}}$ = 0.28	${R_{\text{sa}}}$ = 0.06	${L_{\text{sa}}}$ = $0.22\cdot 10^{-3}$
${C_{\text{sp}}}$ = 3.72	${R_{\text{sp}}}$ = 0.987	
${C_{\text{sv}}}$ = 111.11	${R_{{\text{sv}}}}$ = 0.0113	
${C_{\text{pa}}}$ = 0.76	${R_{\text{pa}}}$ = 0.023	${L_{\text{pa}}}$ = $0.18\cdot 10^{-3}$
${C_{\text{pp}}}$ = 5.8	${R_{\text{pp}}}$ = 0.0894	
${C_{\text{pv}}}$ = 25.37	${R_{\text{pv}}}$ = 0.0056	
${C_{\text{la}}}$ = 19.23		
${C_{\text{ra}}}$ = 31.25		

At the beginning of every iteration step of Eq. (3.5), $D_m$, $D_u$ and $D_l$ are determined according to Eqs. (2.18)–(2.20), before *D* is set equal to one of them according to Eq. (2.17). Equation (2.18) is discretized by 2nd order backward finite differences, whereas Eqs. (2.19) and (2.20) are solved by backward Euler. The parameters $\lambda$, $\gamma _l$ and $\gamma _u$ are found in Table 4.

**Table 4:** Parameter values for the valve model consisting of Eqs. (2.16)–(2.20), which are employed in the circulation of Fig. 3. The values of the two pressure dependent resistances are ${k_{\text{ao}}}$ = 3.75e−4 s mL^−1^ and ${k_{\text{pv}}}$ = 1.4e−3 s mL^−1^.

${\lambda_{{\text{ao}}}}$ = 5000 $s^2$	${\gamma_{{\text{u,ao}}}}$ = 1000 *s*	${\gamma_{\text{l,ao}}}$ = 1000 *s*
${\lambda_{{\text{mi}}}}$ = 30,000 $s^2$	${\gamma _{\text{u,mi}}}$ = 1000 *s*	${\gamma_{\text{l,mi}}}$ = 1000 *s*
${\lambda_{{\text{pv}}}}$ = 2000 $s^2$	${\gamma_{\text{u,pv}}}$ = 1000 *s*	${\gamma _{\text{l,pv}}}$ = 1000 *s*
${\lambda_{{\text{tr}}}}$ = 30,000 $s^2$	${\gamma_{\text{u,tr}}}$ = 1000 *s*	${\gamma_{\text{l,tr}}}$ = 1000 *s*

Once *D* is determined for all four heart valves at step *k*, ${\mathbf{M}}^k$, ${\mathbf{A}}^k$ and ${\varvec{\varphi }}^k$ are assembled and the linear system is solved for ${\varvec{\omega }}^{n+1,k+1}$. Initial conditions for the four valve states, pressure and flow rate values can be found in Table 5.

**Table 5:** Initial conditions of the hydraulic circuit for pressure and flow rate values as well as the four valve states. The initial flow rate in and out of compliance vessels is zero.

	*p* [mmHg]	*q* [mL s^−1^]	*D*
lv	5.75	0.00	0.00
sa	83.45	5.68	
sp	83.11	80.87	
sv	3.29	70.95	
ra	2.49	17.39	1.00
rv	2.44	0.00	0.00
pa	11.01	6.60	
pp	10.86	55.38	
pv	5.91	28.61	
la	5.75	0.86	1.00

### Coupling Mechanics and Circulation

Electrophysiology, activation, mechanics and circulation require different temporal resolutions. To capture the fast dynamics of the steep propagation front a small timestep for electrophysiology and activation is used ($\tau _{\text{e}} = 0.05 \; {\text{ms}}$), whereas for the mechanics and the circulation larger timesteps are possible ($\tau _m = \tau _c = 1 \; {\text{ms}}$). Local load steps for the mechanics are performed in between two timesteps ($\tau _{\text{ls}} = 0.5 \; {\text{ms}}$). The temporal evolution is outlined by Algorithm 3.1.

Coupling between the mechanics and the body circulation is achieved *via* ventricular pressure and volume.^19^ Therefore, the volume of the finite element mechanics model is conserved as well. Based on our experience, only an implicit coupling is stable during all phases of a heartbeat. An implicit scheme is obtained by applying the same pressure boundary conditions ${\mathbf{p}} = [p_{\text{lv}} \quad p_{\text{rv}}]^T$ inside the left and right ventricles on both the mechanical model and the body circulation. By minimizing $||{\mathbf{R}}||_\infty = ||{\mathbf{V}}_{fe} - {\mathbf{V}}_{circ}||_\infty$ where ${\mathbf{V}} = [V_{\text{lv}} \quad V_{\text{rv}}]^T$ contains both ventricular volumes, the pressure boundary conditions are updated through Newton’s method. Thus, the Jacobian matrix reads

3.7 $$\begin{aligned} {\mathbf{J}}_{R} = \frac{\partial {\mathbf{R}}}{\partial {\mathbf{p}}} = \frac{\partial {\mathbf{V}}_{fe}}{\partial {\mathbf{p}}} - \frac{\partial {\mathbf{V}}_{\text{circ}}}{\partial {\mathbf{p}}}, \end{aligned}$$

where the derivatives of ${\mathbf{V}}_{fe}$ and ${\mathbf{V}}_{\text{circ}}$ are determined by pressure perturbation. For the results presented here, we have altered pressure values by $10^{-3} \; {\text{mmHg}}$. Perturbation of the mechanical model is based on the previously computed linearization of ${\mathbf{P}}({\mathbf{u}})$. Eventually, Newton iterations

3.8 $$\begin{aligned} {\mathbf{p}}^{k+1} = {\mathbf{p}}^{k} - ({\mathbf{J}}_R^k)^{-1} {\mathbf{R}}^k \end{aligned}$$

with step *k* are performed as long as $||{\mathbf{R}}||_\infty > \varepsilon _n$ is larger than a defined maximum error of $\varepsilon _n = 10^{-5} \; {\text{mL}}$ . Algorithm 3.1 explains the temporal solution algorithm structure of the framework.

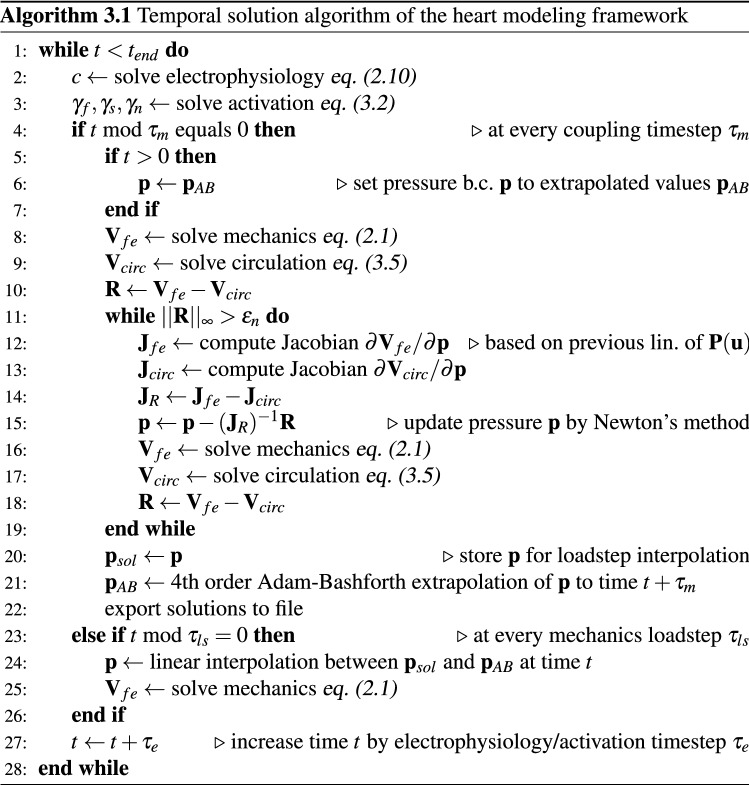



## Validation and Results

All simulation results presented here have been performed on the Euler cluster at ETH Zurich. They were executed on eight Hewlett-Packard BL460c Gen8 nodes, each with 128 GB Ram and two 12-core Intel Xeon E52697v2 processors (2.7 Ghz nominal, 3-3.5 GHz peak); that is, in total on 192 cores. With 160 k elements it took 16.43 hours to simulate one heart beat of 800 ms.

### Validation with Healthy Heart Model

Validation of the whole framework can be done in different ways, for example by comparing contraction patterns from imaging data or by comparing with electrophysiological and flow measurements. However, the range of measurements among individuals is large and therefore our framework shall reproduce common observations, e.g. by how much the distance between base and apex shortens, the wall thickens and the apex twists.

**Figure 4:**
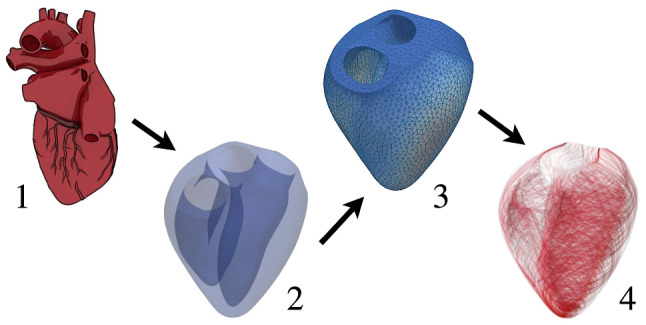
State 1: original geometry provided by Ref. [1]. State 2: the strong muscle structure in the original geometry is extracted. State 3: a tetrahedron mesh is deployed. State 4: fiber and sheetlet direction fields are created.

Most heart geometry measurements are noisy and vary greatly in size. Thus, this framework uses idealized geometrical data, which are very detailed.^1^ The geometry is prepared for simulations according to Fig. 4. As seen in the first state of Fig. 4, raw heart data are detailed and contain many features not required for our studies. From the first to the second state, features such as the great vessels, both the atria, coronary arteries, heart valves and papillary muscles are removed. The final spatial domain, consisting only of the strong ventricular muscles, can be prepared for finite element discretization; it is divided into tetrahedra using GMSH (www.gmsh.info). Three differently sized meshes with 160 k, 250 k and 500 k elements are deployed. While the solid mechanics solutions obtained with 160 k elements hardly differ from those obtained with higher resolution, the electrophysiological problem remains under-resolved with 160 k elements. However, at low resolution the coefficients $\sigma _f$ and $\sigma _s$ of the conductivity tensor ${\mathbf{G}}$ can be adjusted such that the propagation speed of the electric signal is in very good agreement with reality. Therefore, the 160 k mesh was used for the subsequently presented results. Transition from the mesh to fiber and sheetlet fields (state 3 to 4 in Fig. 4) is based on the algorithm described in “Fiber and Sheetlet Directions” section. Fiber angles vary between − 60° to + 60° from both endocardia to the septum and epicardium.

Insulation boundary conditions are applied to the electrical potential, the ionic model and the activation. Boundary conditions for the mechanics are set as follows. On both endocardial surfaces pressure boundary conditions, i.e., ${\mathbf{P}} \cdot {\varvec{\nu }} = p \, {\varvec{\nu }}$, are applied, where *p* comes from coupling with the circulation. At the base, around the four heart valve openings, where the great vessels are attached to the heart, Robin boundary conditions, i.e., ${\mathbf{P}} \cdot {\varvec{\nu }} = \alpha \, {\mathbf{u}}$ with $\alpha = \alpha _b = 750 \; {{\text{mmHg}}\,{\text{cm}}^{-1}}$, are enforced. In order to imitate surrounding tissue such as the lungs, Robin boundary conditions with $\alpha = \alpha _e = 0.75 \; {{\text{mmHg}}\,{\text{cm}}^{-1}}$ are employed on the epicardium as well.

**Figure 5:**
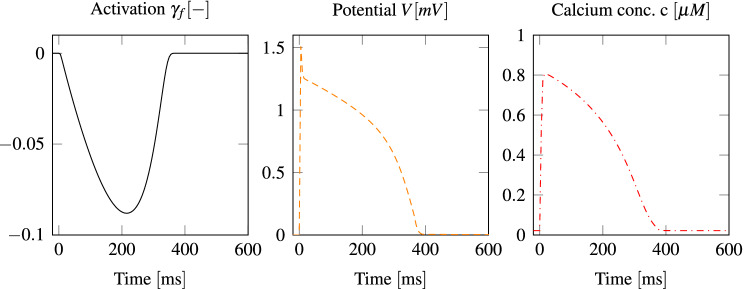
Local temporal evolution of the potential *V*, the normalized calcium concentration *c* and the activation level $\gamma _f$. The unit micromolar $\; {\mu {\text{M}}}$ refers to $\; {10^{-6} \, {\text{mol}} \, {\text{L}}^{-1}}$.

Before the actual simulation starts, the heart is preloaded with initial pressure values, that is, $5.7 \; {\text{mmHg}}$ inside the left ventricle and $2.4 \; {\text{mmHg}}$ in the right one. This preloading requires about 50 load steps causing a volume increase of $25 \; {\text{mL}}$ and $23 \; {\text{mL}}$ in the left and right ventricles, respectively. Every heartbeat lasts $800 \; {\text{ms}}$ and is initiated by an electric wave traveling through the domain; it spreads within $60 \; {\text{ms}}$ through the entire ventricles. Subsequently, we initiate the signal in a simplified fashion by an applied electric current $I_{\text{app}}$ (see Eq. (2.10)), which is applied in the lower third of both endocardial surfaces.

**Figure 6:**
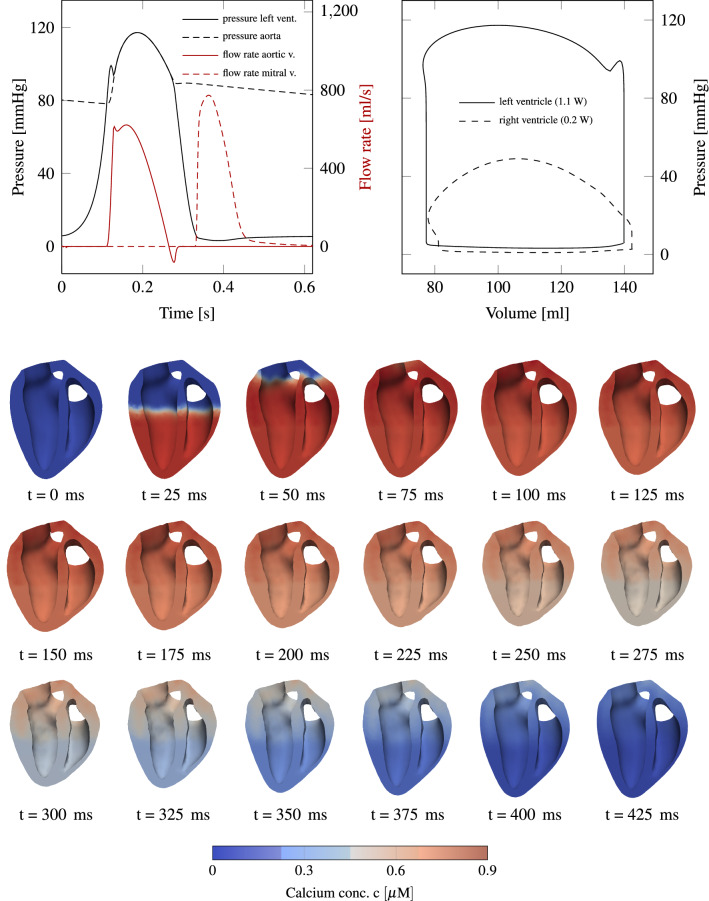
Top left: pressure and flow rate evolution for left ventricular variables. Top right: p-V loop of both ventricles. The work done per heart-beat is equal to the area inside the p-V loops. A total power output of 1.3 W^18^ is in good agreement with measurements. Below a sequence of slices through the left and right ventricles for the first 420 ms of a heartbeat is shown. Color indicates how the calcium concentration spreads through the domain.

Figure 5 presents the evolution of the potential *V*, the normalized calcium concentration *c* and the activation level $\gamma _f$ at a spot inside the left myocardium at half height. At other coordinates the curves would qualitatively be similar, but shifted in time. Results from the lumped parameter model are shown at the top of Fig. 6. Depending on the initial conditions of the model, the results change from one heartbeat to the next one, until they have converged. In our case, this takes usually two to three heartbeats. On the left side of Fig. 6, temporal pressure and flow rate evolution indicates a qualitatively physiological blood flow behavior in comparison to measurements.^4^ In addition, flow rate overshoots are visible as a result of the valve modeling and the phase of refilling through the mitral valve is rather short. On the right side of the figure, p-V loops of both ventricles are drawn. The total power output of the heart model is about 1.3 W, which is in good agreement with measurements.^18^

In order to validate the contraction pattern of the heart model with phenomenological measurements, Fig. 6 displays a sequence of slices through the heart such that both ventricles are seen from inside. The color indicates the calcium concentration spreading through the domain. It can be seen how the heart shortens and how the wall thickness increases, as reported by others.^23,39^ Three phenomenological values, which are appropriate for validation of the ventricular contraction pattern, are the base-apex shortening, wall thickening and base-apex twist.

**Figure 7:**
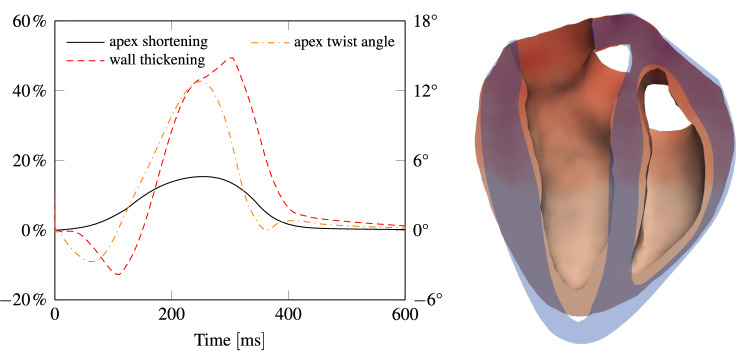
Left: temporal evolution of apex shortening, wall thickening and apex twist, which peak at about 15 %, 45 % and 12°, respectively. Right: cut through the heart, highlighting apex shortening and wall thickening.

The plot on the left of Fig. 7 depicts apex shortening, apex twist and wall thickening as function of time. The right side of Fig. 7 exhibits a cut through the heart before and during contraction, highlighting apex shortening and wall thickening.

### Results with Pathological Heart Model

**Figure 8:**
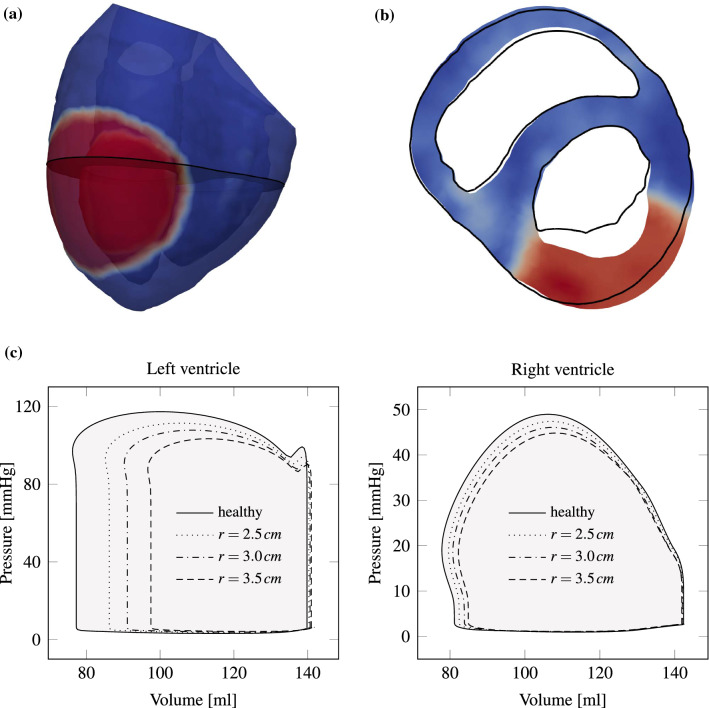
Pathological heart model with myocardial infarction. (a) Transparent image with region of infarction in the left ventricle visible in red ($\gamma _f$ close to 0), whereas healthy tissue is shown in blue ($\gamma _f < 0$). (b) Slice (visible as gray surface in (a)) through the pathologic heart model with black lines indicating the perimeter of a systolic healthy heart as a reference. It can be observed how the diseased tissue dilates more. (c) Left and right ventricular p-V loops are shown for a healthy (filled gray) and various diseased heart models. For an infarction radius of 3.5 cm, power output decreases by almost 50%, whereas for a radius of 2.5 cm the reduction is about 20%.

We consider a pathological heart suffering from coronary heart disease. Thus, the myocardium’s ability to contract during systole is locally reduced, and Eq. (2.8) is adjusted by setting the activation level $\gamma _f$ to zero in a specified region. Figure 8a depicts in red the damaged region of 3 cm radius imposed on the anterior left ventricle. Figure 8b shows a cut through both heart chambers and the myocardial infarction is marked dark red. Black lines indicate the perimeter of a systolic healthy heart. It can be observed how the affected tissue bends outward.

Myocardial infarction not only changes the contraction pattern, but also the cardiac output. Figure 8c makes this change visible by comparing the p-V loops of a healthy heart with those of diseased ones. Since the infarction region is in the left ventricle, the right ventricle’s stroke work hardly changes for all three diseased cases. Left ventricular stroke work for an infarction radius of 3.5 cm decreases by almost 50%, whereas for a radius of 2.5 cm, the reduction is only 20%.

### Results with External Actuation

Force patches (see Fig. 1) are modeled by Dirichlet boundary conditions for Eq. (2.1). For simplicity, two patches with spatially uniform displacement vectors ${\varvec{\zeta }}_1\left( {\mathbf{X}},t\right)$ and ${\varvec{\zeta }}_2\left( {\mathbf{X}},t\right)$ are introduced on the circular boundaries $\varGamma _{D_1}$ and $\varGamma _{D_2}$, respectively, which are defined withing the anterior and posterior epicardial surfaces of the left ventricle. Both patches are rigid (i.e., they contract as one), have a radius of $r=2$ cm and their unit motion vectors ${\varvec{\xi }}$ point toward each other, that is, ${\varvec{\xi }}_1 = - {\varvec{\xi }}_2$. Their displacement vectors ${\varvec{\zeta }}_i$ $\left( i \in \{1,2\} \right)$ allow for a smooth variation between time-steps and are given by

**Figure 9:**
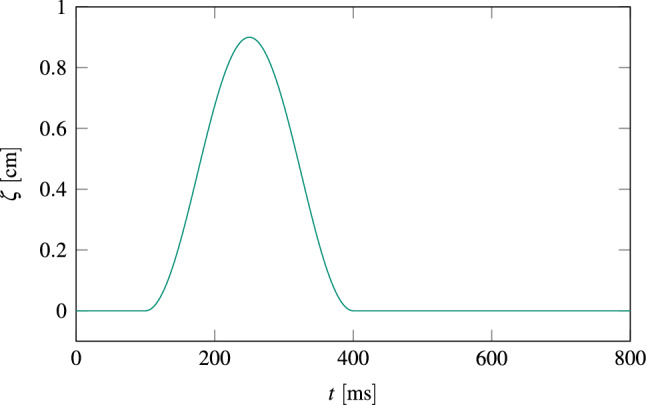
Patch displacement $|{\varvec{\zeta }}|(t)$ according to Eq. (4.1) for a heart beat duration of 800 ms, ${{\hat{t}}}=250$ ms, $T=300$ ms and ${{\hat{\zeta }}}=0.9$ cm.

**Figure 10:**
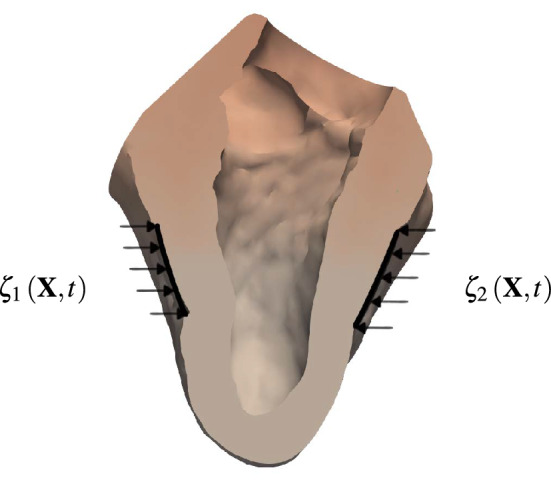
Slice through the left ventricle of a diseased heart during systole with an infarction radius of 3 cm. Two opposite patches ${\varvec{\zeta }}_1\left( {\mathbf{X}},t\right)$ and ${\varvec{\zeta }}_2\left( {\mathbf{X}},t\right)$ assist the heart’s contraction. The arrows on the left and right pointing inwards indicate the applied force direction.

4.1 $$\begin{aligned} {\varvec{\zeta }}_i\left( {\mathbf{X}}, t\right) = {\hat{\zeta }}_i \, \sin ^2 \left( \frac{t-({\hat{t}}_i-0.5 T_i)}{T_i}\pi \right) \, {\varvec{\xi }}_{i} \qquad \forall \, {\mathbf{X}} \in \varGamma _{D_i}, \end{aligned}$$

where ${\hat{\zeta }}_i$ is the peak displacement magnitude, ${{\hat{t}}}_i$ the peak time, $T_i$ the impact duration and ${\varvec{\xi }}_{i}$ the unit vector in motion direction. Both patches move synchronously such that $T=T_1 = T_2$ and ${\hat{t}}={\hat{t}}_1 = {\hat{t}}_2$. Their peak displacement magnitudes will be identical as well, that is, ${\hat{\zeta }}_1 = {\hat{\zeta }}_2$. For the two patches presented above, parameters ${{\hat{t}}}$ and *T* are chosen such that the patch motion is synchronous to natural heart activation, i.e., ${{\hat{t}}}=250$ ms and $T=300$ ms. Figure 9 shows the patch displacement $|{\varvec{\zeta }}|(t)$ for a heart beat duration of 800 ms, ${{\hat{t}}}=250$ ms, $T=300$ ms and ${{\hat{\zeta }}}=0.9$ cm. Further, Fig. 10 visualizes the heart’s deformation when the patches meet their peak displacements. In this demonstration, Dirichlet boundary conditions for patches are only applied in perpendicular directions to the centerline of the left ventricle ${\mathbf{k}}$, i.e., sliding of the tissue underneath the patches is allowed in direction of ${\mathbf{k}}$. Therefore the heart muscle can still properly contract along ${\mathbf{k}}$.

**Figure 11:**
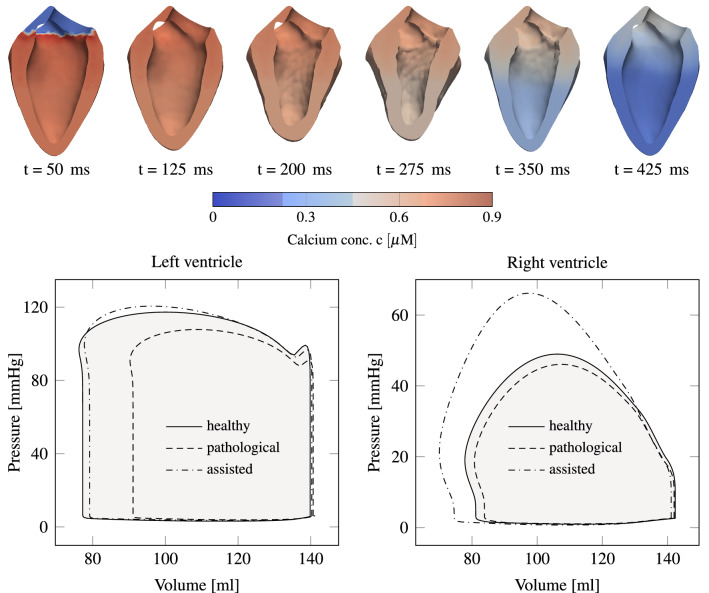
Top: left ventricular deformation during one heartbeat of a diseased model, which is assisted by two opposite patches. The contraction pattern is different compared to healthy conditions (Fig. 6), particularly in the patch regions. The calcium spread is visualized by the color. Bottom: p-V loops for a healthy, a pathological and an assisted pathological heart are presented. The diseased heart has an infarction radius of 3 cm. In order to obtain the same power output as the healthy heart, the pathological heart has to be assisted with a peak displacement of 0.9 cm for both patches.

Under these conditions, to regain a healthy stroke work, we need to impose a peak displacement of magnitude ${\hat{\zeta }}=0.9$ cm; the corresponding p-V loops are shown at the bottom of Fig. 11. Left ventricular results in Fig. 11 are promising regarding stroke work, i.e., a healthy p-V-loop is recovered. However, an unexpected pressure increase can be detected in the right ventricle. It emerges from the heart’s geometry, where the right ventricle is wrapped to some extent around the left one. The top of Fig. 11 presents the deformation of the assisted case during one heart beat. In contrast to Fig. 6, the deformation pattern is most different in the area where patches are attached.

### VAD Sensitivity Analysis

**Figure 12:**
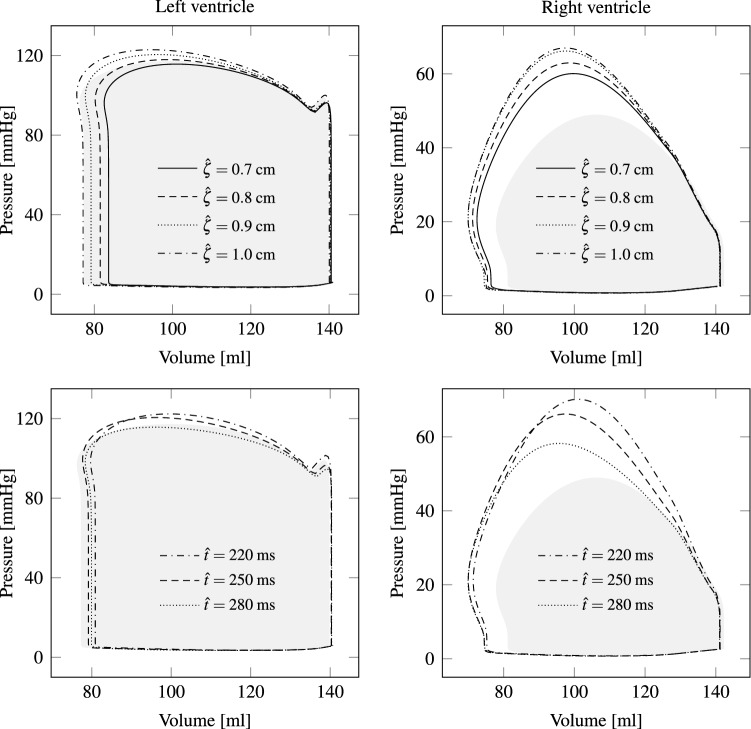
The plots on top demonstrate the sensitivity of the p-V loops on the patch peak displacement. Although both patches are applied on the left ventricle, the right ventricular power output changes by almost the same amount. p-V loops for different peak times ${{\hat{t}}}$ are shown at the bottom. Gray areas illustrate the healthy p-V loops as a reference.

**Figure 13:**
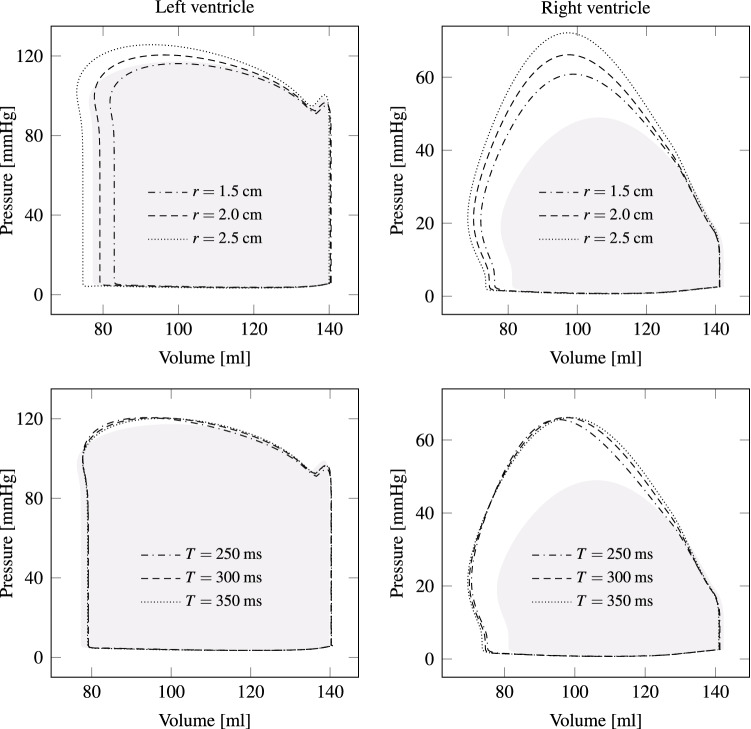
As presumed, a larger patch radius leads to more power output by both ventricles (see top plots). Therefore, patch peak displacement could be reduced by using larger patches, which again results in lower stress in the tissue. The two plots at the bottom indicate that hardly any effect occurs in the left heart chamber when the duration *T* is altered. In the right ventricle, however, the peak pressure decreases with shorter durations *T*. As a reference, healthy p-V loops are shown by gray areas.

In this section we vary the four patch parameters ${\hat{\zeta }}$, ${\hat{t}}$, *T* and *r* and analyze their influence on the p-V loop. From the top of Fig. 12 it can be seen, how the p-V-Loop changes with the displacement magnitude, i.e., for ${\hat{\zeta }}=0.7$, $0.8$, $0.9$, $1.0$ and $1.1 \, {\text{cm}}$. Despite the small changes, a considerable difference in stroke work and pressure values can be observed. Next, the sensitivity of the p-V-loop is analyzed by choosing different peak times ${{\hat{t}}}$ (see bottom of Fig. 12). While the left heart chamber is not much affected, the right ventricular peak pressure and stroke volume decrease with increasing ${{\hat{t}}}$. Thus, overloading the right ventricle may be reduced by adjusting the peak time ${{\hat{t}}}$ appropriately. Here, the patches on the epicardium have circular shape, which may not be optimal. Nevertheless, the patch radius is varied in order to study the influence on the p-V-loop. The top left plot of Fig. 13 demonstrates that a larger patch radius yields more stroke work by the left ventricle. A similar response is observed for the right ventricle.

Eventually, the period *T* in the temporal patch activation function, i.e., in Eq. (4.1), is changed. From the bottom of Fig. 13 hardly any effect can be observed for the left ventricle, whereas the right peak pressure slightly increases for longer durations *T*.

## Discussion and Conclusion

The objective of this paper is to propose a modeling framework for the human heart, which allows for investigations of external actuation *via* force patches attached to the epicardium. The framework consists of a detailed FEM model for the structural mechanics, electrophysiology and actuation, which is coupled to a simplified hydraulic network model representing pulmonary and systemic blood flow circulations. For the latter we introduced a novel valve model, which accounts for a pressure gradient dependent dynamics with typical opening and closing times.

A nonlinear anisotropic Holzapfel–Ogden material law accounting for fiber and sheetlet orientations, and an active strain model responding to the signal of a sophisticated electrophysiological model, lead to very realistic deformations. This has been demonstrated for a healthy heart model. We showed that the computed base-apex shortening, apex-twist and wall thickening are within the expected ranges. The twist first decreases by about 3° and as soon as the outflow valves open, it increases to about 12°. This is in very good agreement with measurements.^22,40,49^ Wall thickening first decreases by about 12%, before it increases up to 45%, as reported similarly in Ref. [23, 39]. The apex shortens from the beginning of activation and the shortening peaks at 15%, which is consistent with observations.^23^ Moreover, predicted pressure and flow rate evolutions are in good agreement with measurements. Of course, this sort of validation is only conclusive to some extent. However, if a model matches these observations, it is likely to reproduce a good overall contraction pattern.

Simulation studies with a pathological model of a heart suffering from myocardial infarction show (as expected) a dramatic reduction of the induced flow rates and significantly different deformations of the heart muscle. Computational results with external actuation using two opposed force patches applied on the epicardium of the diseased heart predict left ventricular pressure and volume changes which are in very good agreement with those of the healthy heart. The right ventricular pressure, however, rises above the desired peak value with this simple actuation scenario. Sensitivity studies with different patch sizes, peak displacements of the patches, peak displacement times, and different impact durations show that the right ventricular power output can be controlled. The gained knowledge can be used to adjust such patch configurations. It is emphasized that a healthy p-V-loop is not the only objective. For example, tissue stress and fatigue, as well as valve and papillary muscle deformation have to be taken into account. And of course it is crucial that the interface between patches and epicardium does not damage the tissue. However, in line with the focus of the paper, this study only serves to demonstrate the potential of the presented computational framework to perform optimization studies, and no attempt has been made yet to find a better combination of parameters, patch geometry and motion. Nevertheless, already the presented results are encouraging for future external VAD developments, and they demonstrate the usefulness of this computational model.

The main limitations of the modeling framework are uncertainties regarding geometry, distribution of material properties and of other parameters. They have been chosen based to the best of our knowledge relying on literature data. Especially among pathological hearts the variation is huge, and it is a separate challenge to retrieve complete descriptions. All presented simulation studies used the same basic heart geometry, and only parameters regarding patches and their actuation have been varied. Further sensitivity studies would be of interest and are planed in the future. There is a demand of personalizing the modeling of human pathophysiology,^26,35^ and it is important to notice that the devised framework can be applied to investigate patient-specific or animal hearts, provided geometry, material properties and all other relevant parameters are available. Another limitation is the simplified flow model. Therefore it is not directly possible to take into account effects of damaged heart valves or the impact of force patches on them. At this point, a further shortcoming is the force patch boundary condition, which only accounts for the force component normal to the epicardium, i.e., tangential forces are ignored. Further, the problem that patches may slide off the slippery surface cannot be addressed, interactions between patches and coronary vessels are ignored, and no conclusions regarding long term tissue damage introduced by the force patches are possible. Also, at this point, independent actuation is implemented; in the future synchronization with the electrical pulses is envisioned.

Despite several limitations, the framework is of significant value for future investigations and optimization of external VADs with force patches, as demonstrated by the computational studies in this paper. In addition, it can be used to study different disease scenarios and/or various assist device designs. On one hand, it can be expected that it will help to reduce the number of required *in vivo* experiments, and on the other hand it allows for investigations which are not even possible *in vivo*. Due to its flexibility, the presented framework has the potential to become an integral part of patient-specific applications. Extraction of geometrical and material information, estimation of further parameters, and uncertainty assessment would be other components of such a much bigger framework.

Potential improvements of the presented computational framework include local modifications of the conductivity tensor in pathologic regions, a completely incompressible treatment of the elasticity by a mixed formulation approach, and atrial contraction by an additional compliance in the hydraulic circuit. Further, a resolved flow field in the two ventricles would be of interest in some cases, e.g. if heart valve functions become part of the investigations. And after all, there exists potential to improve the computational efficiency, which is of particular interest for parameter and optimization studies.

Regarding device development, further sensitivity studies would be of great interest, and optimization not only of patch location, patch displacement peak, displacement peak time and patch size, but also of patch shape and different actuation patterns. In addition to p-V curves, other objectives will be considered for optimization. These will include constraints for patch induced stress and deformation in the valve regions. Alongside computational studies, also ex- and *in vivo* experiments are planned. The intermediate goal is a device for emergency situations during open heart surgery.
